# The CBM-opathies—A Rapidly Expanding Spectrum of Human Inborn Errors of Immunity Caused by Mutations in the CARD11-BCL10-MALT1 Complex

**DOI:** 10.3389/fimmu.2018.02078

**Published:** 2018-09-19

**Authors:** Henry Y. Lu, Bradly M. Bauman, Swadhinya Arjunaraja, Batsukh Dorjbal, Joshua D. Milner, Andrew L. Snow, Stuart E. Turvey

**Affiliations:** ^1^Department of Pediatrics, British Columbia Children's Hospital, The University of British Columbia, Vancouver, BC, Canada; ^2^Experimental Medicine Program, Faculty of Medicine, The University of British Columbia, Vancouver, BC, Canada; ^3^Department of Pharmacology and Molecular Therapeutics, Uniformed Services University of the Health Sciences, Bethesda, MD, United States; ^4^Laboratory of Allergic Diseases, National Institute of Allergy and Infectious Diseases, National Institutes of Health, Bethesda, MD, United States

**Keywords:** CBM complex, CARD11, MALT1, BCL10, combined immunodeficiency, severe combined immunodeficiency, primary atopic disease, BENTA

## Abstract

The caspase recruitment domain family member 11 (CARD11 or CARMA1)—B cell CLL/lymphoma 10 (BCL10)—MALT1 paracaspase (MALT1) [CBM] signalosome complex serves as a molecular bridge between cell surface antigen receptor signaling and the activation of the NF-κB, JNK, and mTORC1 signaling axes. This positions the CBM complex as a critical regulator of lymphocyte activation, proliferation, survival, and metabolism. Inborn errors in each of the CBM components have now been linked to a diverse group of human primary immunodeficiency diseases termed “CBM-opathies.” Clinical manifestations range from severe combined immunodeficiency to selective B cell lymphocytosis, atopic disease, and specific humoral defects. This surprisingly broad spectrum of phenotypes underscores the importance of “tuning” CBM signaling to preserve immune homeostasis. Here, we review the distinct clinical and immunological phenotypes associated with human CBM complex mutations and introduce new avenues for targeted therapeutic intervention.

## Introduction

Inborn errors of immunity or primary immunodeficiency diseases (PIDs) are a group of ~350 genetic disorders that are characterized by defects in immune system development and/or function (1). Defining the genetic and molecular basis of these diseases has not only benefitted the affected individuals but has greatly enhanced our understanding of the fundamental factors that regulate human immunity. A powerful example of the value of studying patients with PIDs is nuclear factor kappa B (NF-κB)—a critical transcription factor that facilitates lymphocyte activation, proliferation and survival. Aberrant signaling in the NF-κB pathway is associated with inflammatory diseases (2), malignancy (3), autoimmunity (4), and immunodeficiency (5). With the description of patients with monogenic immune disorders affecting various components of this signaling cascade, we now have an improved understanding of how NF-κB is positively and negatively regulated.

**Figure 1:**
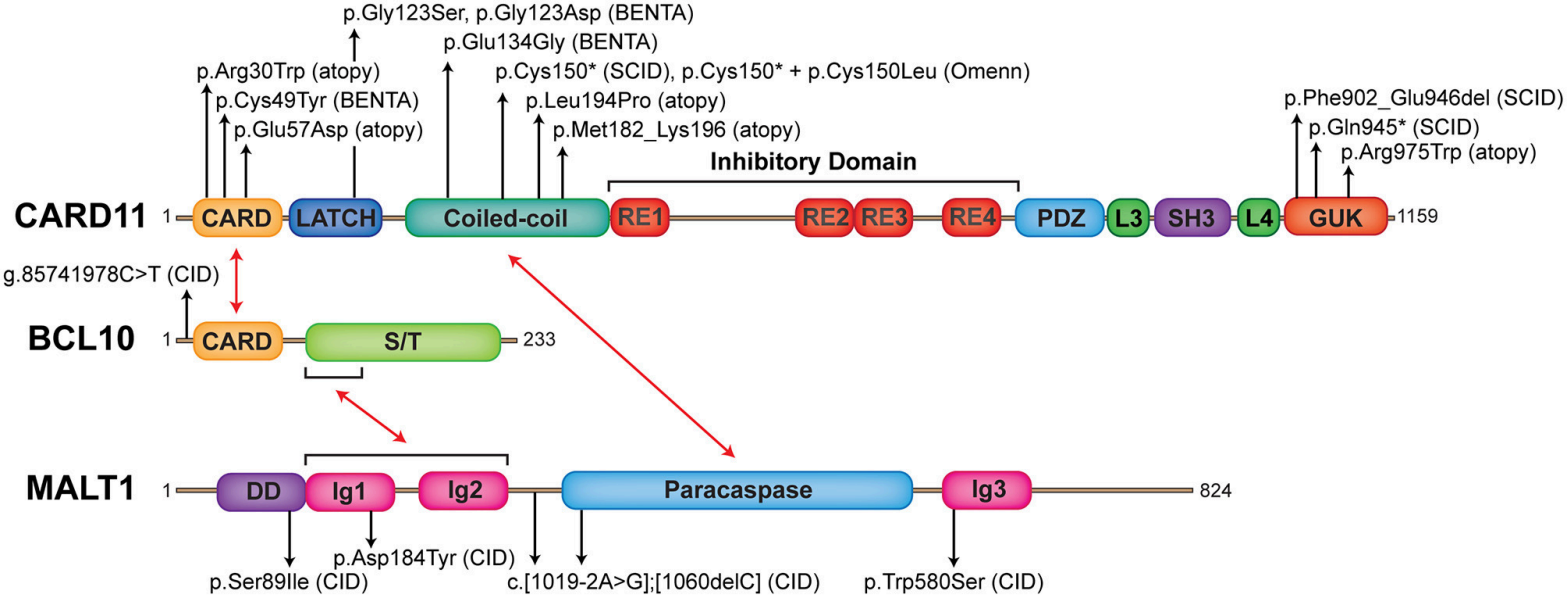
The landscape of human germline mutations causing CBM-opathies. Schematic representation of protein domains found in CARD11, BCL10, and MALT1. Red arrows indicate interactions between domains. Annotated are confirmed mutations causing CBM-opathies and where they localize to on the protein. CARD, caspase recruitment domain; RE, repressive element; L, linker; PDZ, postsynaptic density protein (PSD95), *Drosophila* disc large tumor suppressor (Dlg1), *z*onula occludens-1 protein (zo-1) domain; SH3, SRC homology 3 (SH3); GUK, guanylate kinase domain; S/T, serine/threonine; DD, death domain; Ig, immunoglobulin-like domain.

**Figure 2:**
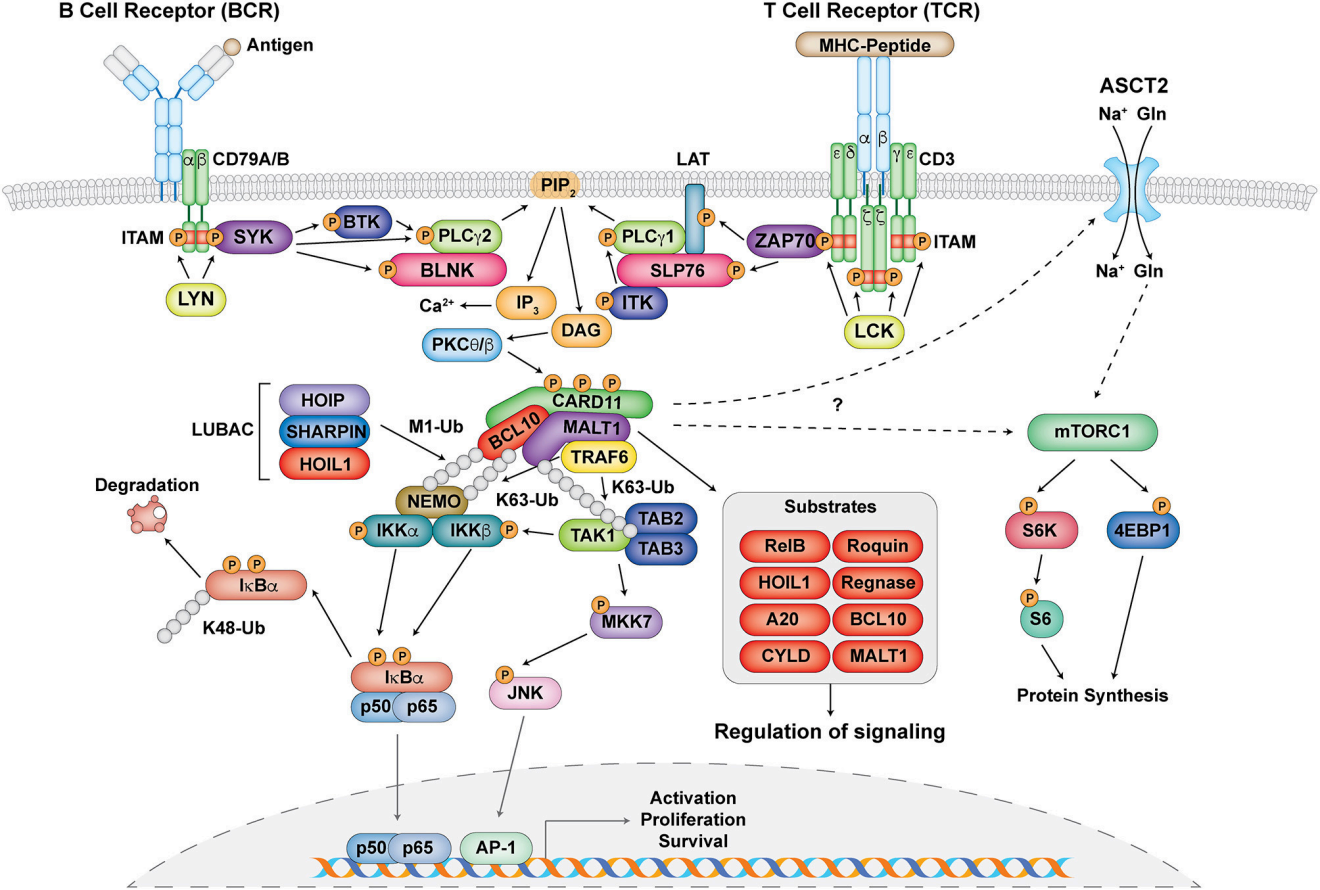
The central role of the CBM complex in BCR- and TCR- signaling. Schematic representation of proximal antigen receptor signaling events in the BCR and TCR, activation and assembly of the CBM complex, and downstream targets and effects of CBM activation. Gray circles represent ubiquitin chains.

In the past decade, the assembly of the caspase recruitment domain family member 11 (CARD11 or CARMA1)—B cell CLL/lymphoma 10 (BCL10)—MALT1 paracaspase (MALT1) [CBM] signalosome complex has emerged as a critical step in the antigen-dependent activation of NF-κB in B and T lymphocytes (6). Major landmarks in the understanding of CBM function first came from oncology. These advances included the characterization of mutant BCL10 and MALT1 proteins caused by chromosomal translocations leading to constitutive/aberrant NF-κB signaling in lymphoma (7), the discovery of CARD11 and its ability to interact with and regulate BCL10 (8), and the identification of mutant CARD11 isoforms affecting the coiled-coil domain that activate NF-κB, constituting ~10% of activated B cell-like diffuse large B cell lymphomas (9, 10). These findings, along with the generation and study of *Card11*^−/−^ (11–13), *Bcl10*^−/−^ (14, 15), and *Malt1*^−/−^ (16) mice, collectively positioned the CBM complex as a central modulator of lymphocyte signaling through the regulation of the NF-κB, c-Jun N-terminal kinase (JNK), and mechanistic target of rapamycin complex (mTORC1) pathways (Figures 1, 2).

The importance of the CBM complex in adaptive immunity was experimentally established by the fact that B and T cells from mice deficient in *Card11, Bcl10*, or *Malt1* all displayed impaired cellular activation and proliferation, aberrant cytokine secretion, and blocks in cell differentiation, resulting in diminished serum immunoglobulin levels. These experimental observations were then validated in the intact human system by the recent discovery of individuals suffering from profound immune defects [i.e., combined immunodeficiency (CID) and severe combined immunodeficiency (SCID)] involving germline loss-of-function (LOF) mutations in *CARD11* (17–19), *BCL10* (20), and *MALT1* (21–23, 24) (Figure 1). While human deficiency of each of the CBM components has some unique defining clinical features (e.g., gastrointestinal inflammation seen in MALT1 deficiency or susceptibility to *Pneumocystis jirovecii* pneumonia (PJP) typical for CARD11 deficiency), as testament to their highly synergistic activities, many phenotypic manifestations are shared across these CBM deficiencies. In particular, some unifying features of CBM PIDs include: CID/SCID occurring in the context of generally normal total B and T cell numbers, a predominantly naïve phenotype in peripheral blood lymphocytes, impaired T cell proliferation, and compromised antigen receptor-induced NF-κB activation.

Recent discoveries have now moved beyond relatively simple LOF mutations, and there is now an interesting spectrum of additional clinical phenotypes attributed to *CARD11* mutations (25), with gain-of-function mutations causing “B cell Expansion with NF-κB and T cell Anergy” (BENTA) disease (26–30), hypomorphic dominant-interfering mutations causing combined immunodeficiency with atopic disease “CARD11-associated Atopy with Dominant Interference of NF-κB Signaling” (CADINS) (31, 32), and loss-of-function mutations with somatic reversion associated with Omenn syndrome (19) (Figure 1).

In this review, we will illustrate the current understanding of CBM-mediated activation of the NF-κB, JNK, and mTORC1 pathways in lymphocytes, and highlight the diverse and rapidly expanding clinical and immunological phenotypes of “CBM-opathies.”

## The CBM complex in antigen receptor signaling

### Proximal antigen receptor signaling

Upon antigen recognition, the CBM complex is primarily involved in signal transduction downstream of antigen receptors leading to the activation of NF-κB, JNK, and mTORC1 in lymphocytes (33–35) (Figure 2). Signaling following B cell receptor (BCR) and T cell receptor (TCR) activation is highly symmetrical and begins with the phosphorylation of immunoreceptor tyrosine-based activation motifs (ITAMs) found on the CD79A/CD79B chains of the BCR and the ζ-chains of the TCR complex by Src family tyrosine kinases LYN and lymphocyte-specific protein tyrosine kinase (LCK), respectively (33, 36). This facilitates the recruitment and phosphorylation of the spleen tyrosine kinase (Syk) family tyrosine kinases SYK (for BCR) and zeta-chain-associated protein kinase 70 (ZAP70) (for TCR) (33, 36) (Figure 2). From here, a collection of adaptor, phospholipase, and kinase proteins come together to form signalosomes, including B cell linker protein (BLNK) and Bruton tyrosine kinase (BTK) for the BCR and SH2 domain containing leukocyte protein of 76 kDa (SLP76), linker of activated T cells (LAT), and IL-2 inducible T cell kinase (ITK) for the TCR. This assembly ultimately culminates in the activation of phospholipase Cγ1 (PLCγ1) for the TCR, PLCγ2 for the BCR, and phosphatidylinositol-4,5-bisphosphate 3-kinase (PI3K) for both (37, 38) (Figure 2).

### CBM assembly

Phosphorylated PLCγ1 and PLCγ2 mediate the hydrolysis of phosphatidylinositol 4,5 biphosphate (PIP_2_) to synthesize the second messengers diacylglycerol (DAG) and inositol-1,4,5-triphosphate (IP_3_) (37, 38). While IP_3_ induces calcium influx, DAG activates protein kinase C (PKC) θ (in T cells) and PKCβ (in B cells) (Figure 2). PKCθ/β act to phosphorylate a series of serine sites along the CARD11 inhibitory domain, the first of several post-translational modifications required for the assembly of the CBM complex (39, 40). CARD11 converts to an open conformation, making it accessible for BCL10-MALT1 binding. BCL10, which constitutively associates with MALT1 through serine/threonine-rich and immunoglobulin-like domain interactions, respectively (7, 41), binds to CARD11 through caspase recruitment domain (CARD)-CARD domain interactions (42) (Figure 1). MALT1 can also bind directly to CARD11 through the interaction of its paracaspase domain and the coiled-coil domain of CARD11 (43). These initial events nucleate the formation of higher order structures consisting of branched BCL10 filaments sheathed with MALT1, allowing for MALT1 oligomerization and activation, and the cooperative recruitment and incorporation of tumor necrosis factor receptor-associated factor 6 (TRAF6) (41, 42).

### Signaling to NF-κB

Canonical NF-κB activation is mediated by the activation of the IκB kinase (IKK) complex, which consists of two catalytic subunits IKKα and IKKβ and a regulatory subunit NF-κB essential modulator (NEMO, also known as IKKγ) (5). After the assembly of the CBM complex, various ubiquitination events occur in order to facilitate the phosphorylation and activation of the IKK complex (35) (Figure 2). MALT1 contains binding motifs for the E3 ubiquitin ligase TRAF6 and acts as a scaffold to facilitate the oligomerization and activation of TRAF6 (44, 45). It is thought that TRAF6 and BCL10, as well as other factors such as the ubiquitin conjugating enzyme UBC13, mediate the K63-linked ubiquitination of various proteins including MALT1 and NEMO (35, 46–48). In addition to K63-linked ubiquitination, M1-linked linear ubiquitin chains are also ligated to NEMO and BCL10 through the linear ubiquitin chain assembly complex (LUBAC), which consists of heme-oxidized IRP2 ubiquitin ligase 1 (HOIL1), HOIL1-interacting protein (HOIP), and SHANK-associated RH domain interacting protein (SHARPIN) (49–51) (Figure 2). These two types of ubiquitination collectively mediate optimal recruitment and phosphorylation/activation of the IKK complex (5).

The phosphorylation of IKKα/β after antigen receptor ligation is thought to be principally mediated by TGFβ-activated kinase 1 (TAK1) and its associated adaptor proteins TAB2/3 (44, 52, 53). It is speculated that since both TAB2/3 and NEMO can specifically recognize K63-linked ubiquitination (47, 54), these two complexes are brought in close proximity to each other, thus facilitating optimal IKK complex activation (34). NF-κB inhibitor alpha (IκBα) in resting cells normally exists in a complex with the NF-κB subunits p50 and p65, which prevents them from becoming activated. The activated IKK complex can phosphorylate IκBα, which causes it to undergo K48-linked ubiquitination and degradation by the proteasome. This allows NF-κB to translocate into the nucleus to initiate target gene transcription (5) (Figure 2).

### Signaling to JNK

Another arm of antigen receptor signaling that the CBM complex mediates is the c-Jun N-terminal kinase (JNK) pathway (Figure 2) [reviewed in (55)]. This process is usually activated by successive phosphorylation events mediated by mitogen-activated protein kinases (MAPKs) (56), with TAK1 also playing a critical role in JNK activation (57, 58). This highlights a cross-talk mechanism in NF-κB and JNK signaling. Although regulation of this process by the CBM complex is not as well understood as the NF-κB pathway, it is thought that TAK1 associates with CARD11-mediated BCL10 oligomers, MKK7 gets recruited, and the selective phosphorylation of JNK2 occurs (59) (Figure 2). This ultimately leads to the accumulation and phosphorylation of c-Jun, which regulates lymphocyte proliferation as part of the AP-1 transcription factor complex.

### Signaling to mTORC1

The mechanistic target of rapamycin (mTOR) kinase is a PI3K-related kinase (PIKK) represented by two distinct catalytic protein complexes, mTOR Complex 1 (mTORC1) and 2 (mTORC2), which have different mechanisms of activation and signaling (60). In particular, mTORC1 can be activated by TCR and CD28 co-stimulation, environmental stimuli such as growth factors and stressors, and nutrients such as amino acids (61). It is critical for regulating T cell growth and proliferation (62) as well as T helper 1 (Th1) and Th17 differentiation (63). Recent studies have demonstrated that the CBM complex regulates TCR-mediated glutamine uptake and the subsequent activation of the mTOR pathway independent of NF-κB (32, 64, 65). Following TCR stimulation, CBM components associate with and mediate the upregulation of the alanine-serine-cysteine transporter 2 (ASCT2) glutamine transporter at the cell surface (32, 64) (Figure 2). MALT1 also has the ability to associate with mTOR, and its paracaspase activity mediates the phosphorylation of the ribosomal protein S6, a target of mTORC1, ultimately impacting metabolic programming (65) (Figure 2). However, the exact molecular mechanism by which the CBM complex links TCR signals to glutamine uptake and the activation of mTOR is unknown and requires further study.

## The CARD family

The CARMA/CARD protein family consists of CARD9, CARD10 (or CARMA3), CARD11 (or CARMA1), and CARD14 (or CARMA2) (66). These scaffold proteins are evolutionarily conserved, structurally homologous, mostly membrane-associated (with the exception of CARD9) and have varying patterns of expression in the body. Mutations in this family of proteins have been implicated in different pathological states, including CARD9 deficiency increasing susceptibility to fungal infections [reviewed in (67)] and CARD14 mutations being linked to increased susceptibility to psoriasis (68, 69) and the skin disease pityriasis rubra pilaris (70).

Each CARD protein participates in its own “CBM” complex with BCL10 and MALT1 in different cell types to facilitate downstream signaling events leading to NF-κB activation (66). CARD11 mediates the activation of lymphocytes by the antigen receptors BCR and TCR and facilitates natural killer (NK) cell activation downstream of ITAM-coupled receptors such as natural killer group 2, member D (NKG2D), NK1.1, Ly49D, and Ly49H (71, 72). CARD14 is expressed in the placenta and skin tissue (69) and can mediate NF-κB activation in keratinocytes possibly downstream of Dectin-1 (73). CARD10 is broadly expressed in non-hematopoietic cells and serves as a link between G-protein coupled receptors (GPCRs) and NF-κB (74). CARD9 is mostly expressed by myeloid cells (macrophages, dendritic cells, neutrophils) and transduces signals emanating from C-type lectin receptors (Dectin-1, Dectin-2, Mincle) and ITAM-associated receptors (FcRγ, DAP12) (67). Although CARD9 deficiency also causes immunodeficiency, this has been covered in detail very recently in the “CARMA Proteins: Playing a Hand of Four CARDs” *Research Topic* (67) and thus, we will be focusing on immune defects caused by mutations in *CARD11*.

## CARD11

### Role of CARD11 in immunity

CARD11 is a ~130 kDa protein originally discovered by bioinformatics screens (8, 75). It is primarily expressed in hematopoietic tissue and lymphocytes and is crucial for antigen receptor signaling (11, 76). CARD11 is a classic scaffold molecule comprised of various defined domains, including CARD, LATCH, coiled-coil (CC), inhibitory, postsynaptic density protein (PSD95), *Drosophila* disc large tumor suppressor (Dlg1), zonula occludens-1 protein (zo-1) (PDZ), SRC homology 3 (SH3), and guanylate kinase (GUK) domains (Figure 1). Initial insight into CARD11 function came from *in vitro* studies on leukemia cell lines, which demonstrated its essential role in NF-κB activation downstream of the TCR complex (77–79). Following this, various CARD11 mouse models were generated, including the *Card11*^−/−^ mouse and CARD11 “unmodulated” (*Card11*^un/un^) mouse (11–13, 80) (compared in Table 1).

**Table 1:** Overview of CBM-deficient mouse models.

**Feature**	***Card11^−/−^***	***Card11^*un*/*un*^***	***Bcl10^−/−^***	***Malt1^−/−^***	***Malt1^*PD*/*PD*^***
**B CELLS**					
**Populations**					
Total	Normal	Normal	Normal	Normal	Normal
FO	Normal	Normal	↓	Normal	Normal
MZ	↓	Normal	↓	↓	↓
B1	↓	↓	↓	↓	↓
GC	ND	ND	ND	↓	↓
**Proliferation**					
IgM	↓	↓	↓	↓	Normal
CD40	↓	↓	↓	↓	ND
CD40L	ND	ND	ND	↓	↓
IgM+CD40	↓	ND	↓	ND	ND
LPS	↓	Normal	Normal	↓ or normal	↓ or normal
IgM+IL-4	ND	↓	ND	↓	↓
CD40+IL-4	ND	↓	ND	↓ or normal	Normal
**Signaling**					
IκBα/NF-κB	↓	↓	↓	↓ or normal	Normal
JNK	↓	↓	↓	↓ or normal	↓ or normal
ERK	Normal	Normal	Normal	Normal	Normal
Tyrosine	Normal	Normal	ND	Normal	ND
Calcium	Normal	Normal	Normal	ND	ND
**T CELLS**					
**Populations**					
Total	Normal	Normal	Normal	Normal	Normal
CD4	Normal	Normal	Normal	Normal	Normal
CD8	Normal	Normal	Normal	Normal	Normal
Treg	↓	↓	↓	↓	↓
Th1	↓	Normal	ND	↑ or normal	↑
Th2	↓	↑	ND	↑ or normal	↑
Th17	↓	Normal	ND	↓ or normal	↑
Tfh	ND	ND	ND	↓	↓
NKT	Normal	ND	Normal	ND	ND
DN3	↓	ND	ND	↓	ND
DN4	↑	ND	↑	↑	↑
DP	ND	ND	↓	Normal	Normal
**Proliferation**					
CD3	↓	Normal	↓	↓	↓
CD3+CD28	↓	↓	↓	↓	↓
P/I	↓	ND	↓	↓	ND
**Signaling**					
IκBα/NF-κB	↓	ND	↓	↓	Normal
JNK	↓	ND	ND	↓	↓ or normal
ERK	Normal	ND	Normal	Normal	Normal
Tyrosine	Normal	ND	Normal	Normal	ND
Calcium	Normal	ND	Normal	ND	ND
**Activation Markers (**α**-CD3**+**CD28)**					
CD25	↓	↓	↓	↓	Normal
CD69	↓	↓	↓	↓	ND
CD40L	ND	↓	ND	ND	ND
OX40	ND	↓	ND	ND	ND
ICOS	ND	↓	ND	ND	ND
CD44	↓	ND	↓	↓	ND
**IMMUNOGLOBULINS**					
**Basal**					
IgM	↓	↓	↓	↓	↓
IgG1	↓	Normal	↓	↓	↑
IgG2a	↓	ND	↓	↓	↓
IgG2b	↓	Normal	↓	↓	↓
IgG3	↓	↓	↓	↓	↓
IgA	↓	ND	↓	↓ or normal	Normal
IgE	ND	↑	ND	ND	↑
**T-dependent**					
IgM	↓	↓	↓	↓	↓
IgG1	↓	↓	↓	↓	↓
IgG2a	↓	ND	↓	ND	ND
IgG2b	ND	ND	↓	ND	ND
IgG3	ND	ND	↓	ND	ND
**Other immune manifestations**	None	Atopy and dermatitis with age	None	None	Spontaneous multi-organ inflammation
References	(11, 12, 81–84)	(13, 17, 85)	(14, 15, 86, 87)	(16, 88–90)	(89, 91–93)

*Lymphocyte characteristics, immunoglobulin levels, and additional immune features are summarized and compared for Card11^−/−^, Card11^un/un^, Bcl10^−/−^, Malt1^−/−^, Malt1^PD/PD^ mice. ND, no data; ↑, increased levels relative to normal range; ↓, decreased levels relative to normal range; FO, follicular B cell; MZ, marginal zone B cell; GC, germinal center B cell; LPS, lipopolysaccharide; IL-4, interleukin 4; IκBα, NF-κB inhibitor alpha; NF-κB, nuclear factor kappa B; Treg, T regulatory cell; Th, T helper cell; Tfh, T follicular helper cell; NKT, natural killer T cell; DN, double negative; DP, double positive; P/I, phorbol 12-myristate 13-acetate (PMA) / ionomycin; JNK, c-Jun N-terminal kinase; ERK, extracellular signal-regulated kinase; ICOS, inducible T cell costimulatory*.

*Card11*-deficient mice were found to be immunodeficient and displayed defective antigen receptor-induced NF-κB and JNK signaling, impaired B and T cell proliferation, and decreased expression of immune activation markers; however, ERK and p38 activation remained intact. Lymphocyte numbers were generally normal, but some abnormalities were present: decreased B1 and marginal zone (MZ) B cells, decreased Th2 and Th17 cells, and absent regulatory T cells (Tregs) (11, 12, 81–84). In addition, both immunoglobulin levels and antibody responses were impaired. These studies highlighted the essential role of CARD11 in mediating B cell development, Treg development, and antibody production.

The *Card11*^*un*/*un*^ mouse, in contrast, possessed a single ENU mutagenesis-induced point mutation in the coiled-coil domain of *Card11*, which did not impact CARD11 protein expression but conveyed a hypomorphic effect on NF-κB and JNK signaling (13). Similar to the *Card11*^−/−^ mice, *Card11*^*un*/*un*^ lymphocytes displayed impaired proliferation and upregulation of activation markers after stimulation (13). Surprisingly, *Card11*^*un*/*un*^ mice did not exhibit overt pathology but developed spontaneous atopy and dermatitis with age. Accordingly, despite some immunoglobulins being decreased, IgE was significantly elevated (13, 85) and this was paired with elevated Th2 cells, diminished Tregs, and unchanged Th1 and Th17 cells (85). These mice highlighted a possible role for CARD11 in the pathogenesis of allergic disease and tolerance. Indeed, a genome-wide association study identified *CARD11* as a susceptibility locus for atopic dermatitis (94) and it was found that CARD11 is important for both Th2 polarization and allergic airway disease (95, 96).

Of the three CBM components, *CARD11* mutations have been associated with the most diverse phenotypes. Human germline *CARD11* mutations cause a broad (and expanding) range of clinical phenotypes including SCID, CID and atopy, and BENTA—with more being characterized (summarized in Tables 2, **4**–**7**).

**Table 2:** Clinical and laboratory phenotype of human CARD11 deficiency.

**Mutation**	p.Gln945^*^	**p.Phe902_Glu946del Exon 21 del**	**p.Phe902_Glu946del Exon 21 del**	**p.Phe902_Glu946del Exon 21 del**	p.Cys150^*^	p.Cys150^*^+ somatic p.Cys150Leu
Age	6 months	9 months	3 months	6 months	Birth	6 months
Sex	F	F	F	M	M	F
Ethnicity	Central European	Palestinian	Palestinian	Palestinian	Turkish	Turkish
Consanguinity	+	+	+	+	+	+
Functional Impact	Loss	Loss	Loss	Loss	Loss	Loss then moderate restoration
Inheritance	AR	AR	AR	AR	AR	AR
Gene Expression	Normal	Truncated	Truncated	Truncated	↓	Normal
Protein Expression	Truncated	None	None	None	None	Restored
**INFECTIONS**						
**Pulmonary**	+	+	ND	+	+	+
*Pneumocystis jirovecii*	+	+	ND	ND	+	–
CMV	–	–	ND	ND	+	+
Human metapneumovirus	–	–	ND	ND	–	+
Rhinovirus	–	–	ND	ND	–	+
**Blood**	–	–	ND	ND	+	+
*Staphylococcus aureus*	–	–	ND	ND	–	+
*Enterococcus*	–	–	ND	ND	+	+
*Pseudomonas*	–	–	ND	ND	–	+
*Klebsiella*	–	–	ND	ND	+	–
**Eyes (retina)**	–	–	–	–	–	+
CMV	–	–	–	–	–	+
**Meningitis**	–	+	–	+	–	–
**CLINICAL MANIFESTATIONS**						
Failure to thrive	–	–	+	ND	–	–
Eczema	–	–	ND	ND	–	+
Erythroderma	–	–	ND	ND	–	+
Lymphadenopathy	–	–	ND	ND	–	+
Hepatosplenomegaly	–	–	ND	ND	–	+
Dyspnea	–	+	ND	ND	–	–
Tachypnea	+	–	ND	ND	–	–
Microcephaly	–	–	ND	ND	+	+
Developmental delay	–	–	ND	ND	+	+
Congestive heart failure	–	–	ND	ND	–	+
Seizures	–	–	ND	ND	+	–
**THERAPY**						
**Transplantation**	+	+	–	–	–	–
Successful	+	+	N/A	N/A	N/A	N/A
**IVIG**	–	+	ND	ND	+	+
**Anti-microbial prophylaxis**	+	+	ND	ND	ND	ND
TMP/SMX	+	+	ND	ND	ND	ND
**IMMUNOSUPPRESSION**						
**Pre-transplantation**	+	ND	–	–	–	–
Treosulfan	+	ND	–	–	–	–
Fludarabine	+	ND	–	–	–	–
Alemtuzumab	+	ND	–	–	–	–
**Post-transplantation**	+	ND	–	–	–	–
Cyclosporine	+	ND	–	–	–	–
Mycophenolate mofetil	+	ND	–	–	–	–
**Outcome**	Alive	Alive	Dead	Dead	Dead	Dead
**Cause of death**	N/A	N/A	Respiratory failure	Respiratory failure	Sepsis	Interstitial pneumonia
**LYMPHOCYTES**						
**Total CD19 B cells**	Normal	Normal	ND	ND	Normal	↓ then normal
Naive	↓	↑	ND	ND	ND	ND
Transitional	↑	↑	ND	ND	↑	ND
(Class-switched) memory	↓	↓	ND	ND	↓	ND
**Total CD3 T cells**	Normal	ND	ND	ND	↑	↓ then ↑*↑↑*
CD4	Normal	Normal	ND	ND	Normal	↓ then ↑*↑↑*
CD4 CD45RA	↑	Normal	ND	ND	Normal	Normal then ↓
CD8	Normal	Normal	ND	ND	↑	↓ then ↑*↑↑*
CD8 CD45RA	↑	Normal	ND	ND	ND	ND
Treg	↓	↓	ND	ND	↓	↓
**PROLIFERATION**						
PHA	↓	↓	ND	ND	↓	↓
ConA	↓	ND	ND	ND	ND	ND
PWM	Normal	ND	ND	ND	ND	ND
CD3	↓	ND	ND	ND	ND	ND
CD3+CD28	ND	↓	ND	ND	↓	↓
**IMMUNOGLOBULINS**						
IgG	↓	↓	ND	↓	↓	↓
IgA	↓	↓	ND	↓	↓	↓
IgM	↓	↓	ND	↓	↓	↓
IgE	ND	ND	ND	ND	Normal	↑
**Specific antibodies**						
Tetanus	ND	ND	ND	ND	Negative	Negative
Pertussis	ND	ND	ND	ND	Negative	Negative
Diphtheria	ND	ND	ND	ND	Negative	Negative
*Haemophilus influenzae B*	ND	ND	ND	ND	Negative	Negative
References	(18)	(17)	(17)	(17)	(19)	(19)

*Key molecular, immunological, infectious, and pathological findings for all patients with CARD11 deficiency described to date. ND, no data; ↑, increased levels relative to normal range; ↓, decreased levels relative to normal range; +, present; −, absent*;

* *premature stop; AR, autosomal recessive; CMV, cytomegalovirus; IVIG, intravenous immunoglobulin; TMP/SMX, trimethoprim/sulfamethoxazole; PHA, phytohemagglutinin; ConA, concanavalin A; PWM, pokeweed mitogen*.

#### Biallelic loss-of-function *CARD11* mutations causing SCID

Germline homozygous loss-of-function (LOF) mutations in *CARD11* are associated with SCID (OMIM 615206) (17–19) (Table 2). To date, there have been three reported cases of complete CARD11 deficiency and they were discovered by whole exome sequencing (WES) or directed Sanger sequencing, with mutations localizing to either the CC (19) or GUK (17, 18) domains. Patients were of different ethnicities but all were born to consanguineous parents: a Palestinian girl with p.Phe902_Glu946del mutations/exon 21 deletion (17), a Central European girl with p.Gln945^*^ mutations (18), and a Turkish boy with p.Cys150^*^ mutations (19). The p.Cys150^*^ homozygous boy also had an older sister with the same mutation, but she additionally had a second site somatic reversion (p.Cys150Leu), which led to Omenn syndrome (19).

Patients typically presented within the first year of life (3–15 months) with severe respiratory tract infections/pneumonia caused by *Pneumocystis jirovecii* (PJP) and abnormal serum immunoglobulin levels that progressed to panhypogammaglobulinemia (3/3 patients). Development was generally normal, and patients did not have any overt organ pathology. All patients generally had normal numbers of total B and T cells, κ-deleted receptor excision circle (KREC)/T cell receptor excision circle (TREC) values (where available), and recent thymic emigrants (Table 2). However, detailed immune profiling revealed a developmental block in B cells, with increased transitional B cells and decreased (class-switched) memory B cells (3/3 patients). All patients had severely diminished Tregs (3/3 patients) and a predominantly naïve T cell phenotype (2/2 patients). Functional evaluation of these patients revealed absent/severely diminished lymphocyte activation of NF-κB and decreased T cell proliferation in response to antigen receptor stimulation (3/3 patients).

Fuchs and colleagues described a Turkish girl homozygous for p.C150^*^ who had a somatic second site reversion (p.C150L), which restored some CARD11 protein expression and function in her lymphocytes (19). Consequently, this young girl had a very different clinical course from the fully CARD11-deficient patients. She presented at the age of 5 months with features of Omenn syndrome, including severe eczema that progressed to generalized erythroderma, lymphadenopathy, and hepatosplenomegaly (Table 2). She experienced multiple bouts of sepsis caused by both bacteria and viruses (*Staphylococcus aureus, Enterococcus, Pseudomonas*) and eventually succumbed to viral pneumonia positive for human metapneumovirus, rhinovirus, and cytomegalovirus (CMV). Immune investigations revealed progressive T cell lymphoproliferation, massive infiltration of highly proliferative T cells in the skin, oligoclonal T cell expansion, and elevated IgE. Interestingly, the patient also had some features that overlapped with fully-deficient patients, including progressive panhypogammaglobulinemia and reduced T cell proliferation (Table 2). Surprisingly, this somatic reversion led to only a partial restoration of NF-κB activity without causing a gain-of-function (GOF) effect. It is thought that the somatic reversion occurred only in some founder T cells, giving them a proliferative advantage over the fully CARD11-deficient T cells. In the context of specific immune triggers such as viral infections, these T cell clones expanded, perturbing immune homeostasis in the absence of Tregs, and ultimately caused skin infiltration, progressive eczema and erythroderma, lymphadenopathy, and hepatosplenomegaly. This case highlights the differential requirements for NF-κB signaling between T cell ontogeny and maintenance.

#### Diagnosis and treatment of CARD11 deficiency

The first cases of CARD11 deficiency were diagnosed by WES (17, 18), while the latter two were found by directed Sanger sequencing based on suspicion and similarity to the first two cases (19). CARD11 deficiency should be considered in patients who present in early life with respiratory tract infections most commonly caused by *Pneumocystis jirovecii* (PJP) (3/3 patients), impaired B cell development with increased transitional and decreased memory (3/3 patients), predominantly naïve T cells (2/2 patients), impaired T cell proliferation (3/3 patients), and panhypogammaglobulinemia (3/3 patients) (Table 3). Specific testing for NF-κB phosphorylation, IκBα degradation, and IL-2 secretion, while often only available in the research setting, will show decreased/absent response. In the appropriate clinical context, sequencing of the *CARD11* gene should help confirm the diagnosis, but depending on the nature of any genetic variants identified, functional tests of the CBM may be required to make a definitive diagnosis (97).

**Table 3:** “Red flags” suggestive of human CARD11 deficiency (LOF *CARD11* mutations).

**Clinical features**	**Proportion**	**Penetrance (%)**
**Infections (bacterial/viral)**		
*P. jirovecii* pneumonia	3/3	100
Bacterial sepsis (*Enterococcus* etc.)	1/3	33
Viral pneumonia (Rhinovirus etc.)	1/3	33
**LABORATORY TESTS**		
**Cell populations**		
Normal total lymphocyte numbers	3/3	100
↓ Treg	3/3	100
↑ Naïve ↓ Memory B cells	3/3	100
↑ Naïve ↓ Effector T cells	2/2	100
**Response**		
↓ T cell proliferation (PHA, α-CD3/CD28)	3/3	100
↓ NF-κB phosphorylation/IκBα degradation	3/3	100
↓ IL-2 secretion	3/3	100
**Ig**		
Panhypogammaglobulinemia	3/3	100

*Tabulation of findings that may be diagnostic clues for CARD11 deficiency. Included are the proportion of patients where the data is available as well as the percentage of patients with the specific phenotype. ↑, increased levels relative to normal range; ↓, decreased levels relative to normal range*.

In addition, patients presenting with features of Omenn syndrome should also be evaluated for CARD11 deficiency (with possible somatic second site reversion) (19). Although there has only been a single patient described with this phenotype to date, suggestive clinical features include erythroderma, lymphadenopathy, hepatosplenomegaly, T cell lymphocytosis, diminished naïve T cells, eosinophilia, and elevated IgE. Analysis of TCR rearrangement would possibly detect oligoclonality. Sequencing *CARD11* in cells from different sites of the body may show different genotypes (LOF vs. LOF+somatic reversion).

In general, CARD11 deficiency is fatal within the first 2 years of life (3/5 died). Infection-related respiratory failure was the most common cause of death (2/5 patients) (17) and one died of sepsis (19). The two patients who survived (17, 18) received successful hematopoietic stem cell transplants. One survivor received bone marrow-derived stem cells from a human leukocyte antigen (HLA)-identical brother who was a heterozygous carrier of the *CARD11* mutation (17), emphasizing that *CARD11* haploinsufficiency is not pathological. The second survivor received peripheral blood hematopoietic stem cells from an HLA-matched unrelated donor (18). Overall, these results indicate that patients with confirmed CARD11 deficiency should be considered for curative hematopoietic stem cell transplantation as soon as possible following diagnosis. While awaiting transplant, CARD11-deficient patients should receive immunoglobulin replacement therapy and PJP prophylaxis.

#### Dominant negative loss-of-function *CARD11* mutations causing CID, atopy, and novel phenotypes

Germline heterozygous LOF mutations in *CARD11* are associated with severe atopic disease and CID with a susceptibility to infections (OMIM 617638) (31, 32). These LOF mutations dominantly interfere with wild-type (WT) *CARD11* and signaling to NF-κB and mTORC1, thus explaining the observed autosomal dominant inheritance pattern. To date, five distinct dominant negative (DN) LOF CARD11 mutations have been linked to disease in 12 patients (Figure 1). Although the cohort of known CARD11 DN patients and associated clinical phenotypes is expanding rapidly (98) (Table 4), the cardinal feature noted in ~90% of patients is severe atopic disease, encompassing symptoms of immediate hypersensitivity (allergic rhinitis, food allergy) and/or allergic inflammation (atopic dermatitis, eosinophilic esophagitis), specific allergens notwithstanding (99). Importantly, as discussed in the “Role of CARD11 in Immunity” section, similar atopic phenotypes were described in unmodulated (*Card11*^un/un^) mice harboring a hypomorphic mutation in *Card11* (13, 85) and *CARD11* has previously been identified as a risk locus for atopic dermatitis in a Japanese genome-wide association study (94).

**Table 4:** “Red flags” suggestive of CADINS disease (DN LOF *CARD11* mutations).

**Clinical features**	**Proportion**	**Penetrance (%)**
**Atopic disease**	**39/44**	**89**
Atopic dermatitis	32/44	73
Asthma	24/44	55
Food allergies	14/44	32
Eosinophilic esophagitis	3/44	7
**Cutaneous viral infections**	**30/44**	**68**
**Respiratory infections**	**30/44**	**68**
Autoimmunity	9/44	20
Neutropenia	6/44	14
Oral ulcers	6/44	14
Lymphoma	3/44	7
**LABORATORY TESTS**		
**Cell Populations**		
↑ Total CD4^+^ T cells	4/40	10
↓ Memory CD4^+^ T cells	9/26	35
Normal total CD8^+^ T cells	41/43	95
↓ Memory CD8^+^ T cells	4/15	27
↓ Total B cells	8/43	19
↓ Class-switched/memory B cells	10/35	29
↓ NK cells	8/43	19
↓ Tregs	2/29	7
↑ Eosinophils	26/40	65
***In vitro*** **responses**		
↓ T cell proliferation	19/31	61
↓ NF-κB phosphorylation/IκBα degradation	11/12	92
Specific antibody response defect	20/41	49
Total antibody response defect	12/42	29
**Ig**		
Panhypogammaglobulinemia	5/44	11
↑ IgE	31/42	74

*Summary of major clinical and immunological phenotypes associated with human germline DN LOF CARD11 mutations causing CARD11-associated Atopy with Dominant Interference of NF-κB Signaling (CADINS) disease. Proportion of patients and penetrance is calculated based on number of patients with available data. Clinical “red flags” for potential diagnosis of CADINS disease are marked in blue. ↑, increased levels relative to normal range; ↓, decreased levels relative to normal range*.

Heterozygous *CARD11* mutations were initially identified by WES in eight patients with severe atopic dermatitis (32). These mutations included three missense mutations encoding p.Glu57Asp, p.Leu194Pro, and p.Arg975Trp and one in-frame mutation encoding a 14-amino acid insertion (p.Met183_Lys196). These patients generally possessed features of recalcitrant atopic dermatitis with elevated serum IgE levels and eosinophilia, with severity waning with age in certain patients. Most patients also presented with respiratory distress associated with recurrent pulmonary infections and pneumonias, as well as viral skin infections (e.g., molluscum, eczema herpeticum). Four additional patients were subsequently described with missense mutations encoding p.Arg30Trp, leading to multiorgan atopy, autoimmunity, and a prominent susceptibility to infections (31).

These mutations affected several different domains of the CARD11 protein, with 2 in the CARD (p.Arg30Trp and p.Glu57Asp), 2 in the CC (p.Leu194Pro and p.Met183_Lys196), and 1 in the GUK (p.Arg975Trp) domain (31, 32). Patient T cells and mutant plasmid-transfected Jurkat T leukemia cell lines demonstrated that each variant impaired TCR-induced NF-κB activation by disrupting WT CARD11 signaling. Subsequent studies of an expanded patient cohort have identified at least 10 additional DN mutations primarily found in the CARD and CC domains, where they are most likely to impede CBM complex assembly by thwarting BCL10 and MALT1 binding and oligomerization (98). In addition to NF-κB blockade, many (but not all) of these mutations also reduced TCR-mediated mTORC1 activation, ostensibly by preventing optimal glutamine uptake through upregulation of the ASCT2 transporter (32). These TCR signaling defects not only resulted in impaired T cell proliferation and induction of cell surface activation markers, but also contributed to a Th2-skewed CD4^+^ T cell phenotype, with enhanced secretion of IL-4 and IL-13 and decreased IFN-γ production. Intriguingly, *in vitro* culture of patient T cells with excess glutamine partially restored T cell proliferation and IFN-γ secretion in the presence of cytokines that trigger NF-κB (e.g., IL-1/TNF) and signal transducer and activator of transcription (STAT3) (e.g., IL-6) activation independent to the TCR (32). These findings suggest that both NF-κB and mTORC1 signaling defects contribute to atopic predisposition and disease pathology, even though diagnostic readouts of impaired mTORC1 signaling (e.g., ribosomal protein S6 phosphorylation) can be variable and difficult to detect experimentally. In contrast to *Card11*^un/un^ mice, the frequency and suppressive function of Tregs is normal in almost all CARD11 DN patients tested to date, suggesting important mechanistic discrepancies between mouse and human (31, 32).

#### Diagnosis and treatment of disease caused by DN *CARD11* mutations

The recent identification of many additional patients harboring CARD11 DN variants provides a clearer picture of the full phenotypic spectrum of disease (98) (Table 4). Clinically, patients with germline DN *CARD11* mutations most often present in early childhood with atopy, cutaneous viral infections, and recurrent respiratory infections. These signs and symptoms occur in an autosomal dominant manner with high penetrance and no gender bias. However, subsets of patients may also present with hypogammaglobulinemia and specific antibody deficiency (SAD), neutropenia, oral ulcers, autoimmunity (e.g., alopecia), or lymphoma. Diagnostic tests include: (i) assaying for a NF-κB signaling defect in response to TCR or PMA stimulation (reduced p65 phosphorylation, IκB degradation) and (ii) sequencing *CARD11* to identify rare/novel variants, with CARD and CC domain variants having the highest likelihood of being pathogenic. Ultimately, functional testing (e.g., Jurkat T cell transfections) is highly recommended to confirm DN LOF activity for any novel variants found.

Based on these collective clinical and experimental findings, we propose to classify this disorder as CARD11-associated Atopy with Dominant Interference of NF-κB Signaling (CADINS) disease. Although more work is required to mechanistically connect faulty CARD11 signaling to the various phenotypes of CADINS disease, defects in both T and B cell function explain CID in these patients and underscore the essential role of CARD11 in governing peripheral lymphocyte differentiation and effective humoral immune responses. Continued mechanistic studies will also inform future clinical management and therapeutic strategies for these patients. Although glutamine supplementation may offer the simplest intervention (see “Novel Therapeutic Insights Emerging From Our Understanding of Human CBM-opathies” section), newer biologics targeting Th2 cytokine signaling (e.g., dupilumab, mepolizumab) or IgE directly (omalizumab) may be useful in ameliorating atopic disease (100). In contrast to CARD11 deficiency, hematopoietic stem cell transplantation should only be considered in the most severe pediatric cases, since symptoms may improve with age.

#### Gain-of-function *CARD11* mutations causing BENTA

B cell Expansion with NF-κB and T-cell Anergy (BENTA) is a congenital lymphoproliferative and immunodeficiency disorder caused by heterozygous GOF *CARD11* mutations (OMIM 616452) (26). The first patient diagnosed with BENTA disease was initially reported in 1971, and presented with splenomegaly and persistent B cell lymphocytosis that worsened with splenectomy and resembled chronic lymphocytic leukemia (CLL) (101). This patient eventually developed monoclonal CLL around age 44 and received a curative hematopoietic stem cell transplant from his sister (26). His two daughters presented with frequent sinopulmonary and ear infections and were found to exhibit splenomegaly and marked B cell lymphocytosis in infancy (26). RNA-Seq analyses of this first patient and his two daughters revealed a novel heterozygous missense mutation (p.Glu134Gly), located in the N-terminal portion of the CC domain of CARD11. A fourth unrelated patient that presented with similar symptoms was simultaneously identified with unique heterozygous missense mutations located in the LATCH domain (p.Gly123Ser) (26). Since the initial description of BENTA disease in these four patients in 2012, GOF *CARD11* mutations associated with BENTA have been identified in >25 additional patients (27–30, 102–104) (Figure 1, Table 5).

**Table 5:** “Red flags” suggestive of BENTA disease (GOF *CARD11* mutations).

**Clinical features**	**Proportion**	**Penetrance (%)**
**Splenomegaly in childhood**	**14/14**	**100**
**INFECTIONS**		
**Otitis media/sinopulmonary**	**21/21**	**100**
EBV	9/21	43
Molluscum contagiosium	8/22	36
**LABORATORY TESTS**		
**Cell populations**		
**B cell lymphocytosis**	**21/21**	**100**
↑ % Naïve mature B (IgM^+^ IgD^+^)	21/21	100
↑ % Immature/transitional B (CD10^+^)	21/21	100
↓ % Class-switched and memory B	21/21	100
**Normal T cells (abs #)**	21/21	100
↑ % DN T cells	6/10	60
**Autoantibodies**	5/21	23
**Autoimmune hemolytic anemia**	4/21	19
***IN VITRO*** **RESPONSES**		
**Naïve B cells**		
Normal proliferation	7/7	100
↓ Plasma cell differentiation	7/7	100
↓ IgG secretion	7/7	100
**T cells**		
↓ Proliferation (α-CD3/CD28)	7/7	100
↓ IL-2 secretion (α-CD3/CD28, mitogens)	7/7	100

*Summary of major clinical and immunological phenotypes associated with human germline GOF mutations in CARD11 causing B cell Expansion with NF-κB and T-cell Anergy (BENTA) disease. Proportion of patients and penetrance is calculated based on number of patients tested with available data. Clinical “red flags” for potential diagnosis of BENTA disease are marked in blue. GOF CARD11 mutations are bolded. ↑, increased levels relative to normal range; ↓, decreased levels relative to normal range*.

The primary hallmark of BENTA disease is polyclonal B cell lymphocytosis in early childhood paired with splenomegaly and lymphadenopathy. Pediatric patients possess excessive accumulation of immature transitional (CD10^+^CD24^hi^CD38^hi^) and mature naïve (IgM^+^IgD^+^) polyclonal B cells, with very low percentages of circulating memory and class-switched B cells. Circulating naïve and transitional B cell counts typically decrease into adulthood, likely reflecting reduced output of immature B cells from the bone marrow. Conversely, patient T cell numbers are usually normal, unless chronic viral infection (e.g., EBV) is present. Histologic analyses of lymphoid tissues reveal follicular hyperplasia with an impressive expansion of naïve IgD^+^ B cells in mantle zones, but normal numbers and distribution of CD3^+^ T cells (26). Aside from selective B cell lymphocytosis, BENTA patients also exhibit features of primary immunodeficiency. All BENTA patients experienced frequent ear and sinus infections in early life, and opportunistic viral infections such as molluscum contagiosum and JC/BK virus are noted in some patients. Chronic EBV infection with moderate viremia is also found in ~50% of BENTA patients (104).

Similar to specific antibody deficiency (SAD) (105), poor humoral immune responses are observed in most BENTA patients in response to T cell-independent vaccines such as pneumococcal and meningococcal polysaccharide vaccines, even with repeated boosts. Some patients also fail to mount lasting protective titers to T cell-dependent conjugate vaccines for pneumococcal bacteria (i.e., Prevnar), varicella-zoster virus (VZV), or measles. Low serum IgM and IgA levels are noted in some patients, with IgG being variable. *In vitro* studies of naïve patient B cells demonstrated impaired B cell differentiation into plasmablasts and long-lived plasma cells, consistent with poor IgG secretion in culture (106). These defects could be explained in part by a failed induction of specific factors required for plasma cell commitment, including BLIMP-1 and XBP-1. Conversely, in mice, ectopic expression of GOF *CARD11* variants in activated B cells promoted the transient expansion of self-reactive plasmablasts and autoantibody production (107). This discrepancy may be due to differences in mouse and human B cell differentiation requirements or may reflect the need for *in vivo* cytokines that were not provided *in vitro*. Nevertheless, profound apoptosis resistance was readily observed in both mouse and human B cells expressing GOF *CARD11* variants and may be the most likely driver of B cell lymphocytosis in BENTA disease. Surprisingly, BENTA patient T cells are generally hyporesponsive in culture with poor proliferation and reduced IL-2 secretion (26). T cell function can largely be rescued by stronger stimulation or IL-2 supplementation *in vitro*, implying a mild state of anergy in BENTA T cells. Although autoantibodies are detected in a few patients, autoimmune disease symptoms are not common in BENTA patients, perhaps reflecting underlying B and T cell differentiation defects.

Most of the germline GOF *CARD11* mutations described in BENTA patients (p.Cys49Tyr, p.Gly123Ser, p.Gly123Asp, p.Phe130Ile, p.Glu134Gly) are also found as somatic GOF *CARD11* mutations in diffuse large B cell lymphoma (DLBCL) and other lymphoid malignancies (9, 108). In fact, knockdown of *CARD11* effectively kills DLBCL cell lines harboring GOF *CARD11* mutations, underscoring the connection between enhanced CBM signaling and B cell growth and survival. Remarkably, these single GOF mutations can disrupt the auto-inhibition of CARD11 conferred by several repressive elements within the inhibitory linker domain (109, 110). This allows CARD11 to adopt an open, active conformation and drive constitutive NF-κB activation via spontaneous aggregation, unimpeded recruitment of BCL10/MALT1, and IKKα/β phosphorylation, in the absence of antigen receptor engagement (26, 28, 109, 110). Indeed, spontaneous CARD11 aggregation and elevated NF-κB signaling is also observed in B and T cells from BENTA patients (26). In addition, the ectopic expression of BENTA-associated *CARD11* mutants in B and T cell lines results in the spontaneous assembly of large protein aggregates including CARD11, BCL10, MALT1, and phosphorylated IKKα/β, which induces constitutive NF-κB signaling independent of antigen receptor ligation (26, 28).

Despite highly congruent signaling pathways emanating from the TCR and BCR, constitutive activation of canonical NF-κB driven by GOF CARD11 mutations in BENTA disease leads to surprisingly distinct functional consequences in B and T cells. However, the mechanisms behind this dichotomy in B and T cell phenotypes remain unclear. Previous studies using conditional transgenic mice offer tantalizing parallels; while B cell-specific expression of constitutively active IKKβ (caIKKβ) promotes survival and proliferation even in the absence of B cell activating factor (BAFF) (111), restricted transgenic expression of caIKKβ renders murine T cells anergic and more susceptible to apoptosis, consistent with poor responses to bacterial infections (112). Collectively, studies of BENTA patients to date indicate that constitutive NF-κB can also lead to combined immunodeficiency, albeit less severe than patients harboring (DN) LOF CARD11 mutations. We therefore speculate that intrinsic B cell defects in BENTA disease most likely contribute to impaired humoral immunity and frequent infections with extracellular bacteria, while mildly anergic T cells could make BENTA patients more susceptible to certain viral infections.

#### Diagnosis and treatment of BENTA

Patients presenting with splenomegaly, selective B cell lymphocytosis, and frequent sinopulmonary infections early in life should raise suspicion for BENTA disease. Sequencing of *CARD11* may find variants, particularly in the LATCH or CC domains. It is recommended that novel variants be confirmed experimentally (e.g., B or T cell line transfections to look for constitutive NF-κB activation) and cross-referenced to reported somatic mutations in lymphoma using Catalogue of Somatic Mutations in Cancer (COSMIC) (113) or related databases of oncogenic mutations.

At present, BENTA patients are clinically managed with supportive therapy, and minimal therapeutic interventions are available. Polyclonal B cell lymphocytosis in BENTA disease may predispose patients to B cell malignancies later in life. However, only two patients with confirmed malignancy have been reported to date: B cell CLL at ~44 years in the original index patient (26), and another with Hodgkin's lymphoma at ~50 years of age. Still, BENTA patients should be regularly monitored for B cell clonal outgrowth using flow cytometry and IgH heavy chain rearrangement analyses. The presence of EBV viremia may also heighten the risk of B cell lymphomagenesis.

Removal of the spleen is generally not recommended, given that circulating B cell counts rose dramatically in 3 patients after splenectomy, and splenectomy itself may put the patient at increased risk for certain bacterial infections (26, 28). One patient with a p.Gly123Asp mutation and an exceptionally high number of peripheral B cells after splenectomy was treated with methotrexate for 4 years to restrain B cell counts and reduce the risk of stroke (28). Rituximab was effective in both this patient and another with respiratory distress and excessive lymphocytic nodules in her lungs. However, the utility of B cell depleting agents for BENTA should be evaluated on a case-by-case basis and may not be necessary as B cell lymphocytosis wanes over time. Intravenous or subcutaneous immunoglobulin therapy has also been administered in a few patients during childhood to control infections. Interestingly, MALT1 protease inhibitors, which specifically constrain CBM signaling output without completely blocking NF-κB activation, could be an attractive targeted treatment option for certain BENTA patients (see Novel Therapeutic Insights Emerging From Our Understanding of Human CBM-opathies section). These inhibitors are currently being explored for treating B cell lymphomas and autoimmune diseases (114–117).

## BCL10

### Role of BCL10 in immunity

BCL10 was originally identified from a recurrent breakpoint (1p22) in mucosa-associated lymphoid tissue (MALT) B cell lymphomas possessing the t(1;14)(p22;q32) translocation, which caused BCL10 to be overexpressed (118). This observation, in combination with the finding that BCL10 could potently induce NF-κB activation (119, 120), highlighted its involvement in NF-κB signaling. BCL10 is a ~27 kDa protein, which contains an N-terminal CARD domain and C-terminal serine/threonine rich region (Figure 1). Through CARD-CARD interactions, BCL10 can oligomerize with other CARD-containing proteins, including CARD9, CARD10, CARD11, and CARD14 (8, 75, 121–123) as well as MALT1 (7) to form various CBM complexes. These complexes collectively regulate both innate and adaptive immune processes in various cell types, although its lymphoid role is the main focus here. Like CARD11, BCL10 also undergoes various post-translational modifications that regulate CBM assembly and signaling and can form high order filamentous structures [reviewed in (124)]. The generation of *Bcl10*^−/−^ mice defined key physiological roles for BCL10, particularly its essential role in antigen receptor signaling (Table 1) (14, 15, 86, 87).

Deletion of *Bcl10* in mice causes partial embryonic lethality (1/3 die) caused by issues with neural tube closure during development (14). Aside from this particular phenotype, *Bcl10*^−/−^ mice were immunodeficient and generally resembled *Card11*^−/−^ mice. They possessed normal total numbers of B and T cells, but decreased numbers of Tregs, natural killer T (NKT), B1, and MZ B cells (15, 86). Lymphocytes lacking Bcl10 failed to activate NF-κB, secrete pro-inflammatory cytokines, proliferate effectively in response to antigen receptor stimulation, and upregulate activation markers (14). Similar to CARD11-deficient mice, *Bcl10*^−/−^ mice also had panhypogammaglobulinemia and impaired T-dependent humoral responses (15).

Interestingly, it was recently demonstrated that BCL10 also contributes to glutamine uptake via the ASCT2 transporter (as mentioned in the “Signaling to mTORC1” section), and together with CARD11 and MALT1, governs Th1 and Th17 polarization independent of the NF-κB pathway (64). However, another group found that BCL10 was dispensable in the phosphorylation of S6 (65). Thus, BCL10 contribution to this pathway is controversial and requires further investigation.

#### Loss-of-function *BCL10* mutations causing combined immunodeficiency

A single case of BCL10 deficiency has been identified in a consanguineous Amerindian boy from Ecuador caused by homozygosity for a germline loss-of-function mutation (OMIM 616098) (20). The patient exhibited features of CID and immune dysregulation. WES discovered a homozygous splice site mutation (g.85741978C>T;IVS1+1G>A) affecting the invariant first nucleotide of intron 1 (donor site for splicing), which led to absent mRNA and protein expression. The patient had a complex clinical course, including respiratory infections positive for influenza A/B, adenovirus, respiratory syncytial virus, gastroenteritis, otitis, oral candidiasis and diaper dermatitis from *Candida albicans* superinfection, recurrent diarrhea positive for *Campylobacter jejuni*, adenovirus, and *Clostridium difficile* at different times, acute gastroenteritis positive for adenovirus, chronic colitis, and suspected encephalitis (Table 6). The patient eventually died due to respiratory failure.

**Table 6:** Clinical and laboratory phenotype of human BCL10 deficiency (LOF *BCL10* mutations).

**Mutation**	**g.85741978C>T;IVS1+1G>A**
Age	6 months
Sex	M
Ethnicity	Amerindian
Consanguinity	+
Functional Impact	Loss
Inheritance	AR
Gene Expression	None
Protein Expression	None
**INFECTIONS**	
**Pulmonary**	+
Influenza A + B	+
RSV	+
Adenovirus	+
**Gastrointestinal**	+
*Campylobacter jejuni*	+
*Clostridium difficile*	+
Adenovirus	+
**Oral**	+
*Candida albicans*	+
**CLINICAL MANIFESTATIONS**	
Failure to thrive	–
Dysmorphic facies	–
Periodontal disease	–
Eczema	–
Enteropathy	+
Bronchiectasis	–
**THERAPY**	
**Transplantation**	–
Successful	N/A
**IVIG**	+
**Anti-inflammatory**	
Mesalazine	+
**Antibiotics**	+
Vancomycin	+
Metronidazole	+
**Other**	Levetiracetam
**Outcome**	Death
**Cause of death**	Respiratory failure
**LYMPHOCYTES**	
**Total CD19 B cells**	↑
Naive	↑
(Class-switched) memory	↓
**Total CD3 T cells**	↑
CD4	↑
CD4 Naïve	↑
CD4 CM	↓
CD4 EM	Normal
CD8	Normal
CD8 Naïve	↑
CD8 CM	↓
CD8 EM	Normal
Treg	↓
**PROLIFERATION**	
PHA	Normal
ConA	Normal
PWM	Normal
CD3+CD28	↓
**IMMUNOGLOBULINS**	
IgG	↓
IgA	↓
IgM	↓
IgE	Normal
Reference	(20)

*Main clinical and immune findings of the single BCL10-deficient patient described to date. ↑, increased levels relative to normal range; ↓, decreased levels relative to normal range; +, present; −, absent; RSV, respiratory syncytial virus; CM, central memory; EM, effector memory*.

Lymphocyte counts were generally normal, but B and T cells mostly displayed a naïve phenotype with an associated reduction in memory B and T cells and a profound absence of Tregs (Table 6). In keeping with the naïve phenotype, the patient also displayed hypogammaglobulinemia. Interestingly, in contrast to murine studies, patient myeloid cells responded normally to innate ligands (125–127), while fibroblasts displayed impaired NF-κB activation in response to Toll-like receptor (TLR)2/6, TLR4, and Dectin-1 stimulation as measured by NF-κB nuclear translocation and cytokine secretion (20). In addition, patient T cells displayed an impaired proliferative response to antigen receptor ligation (but not mitogen stimulation), and this was paired with a significant reduction in the expression of activation markers ICOS and CD25. Contrary to murine studies (14), CD69 expression was upregulated normally by T cells.

#### Diagnosis and treatment of BCL10 deficiency

BCL10 deficiency should be considered if a patient is found to have broad immune defects/CID affecting both innate (fibroblasts) and adaptive immunity (B and T cells), especially if a patient presents with severe inflammatory gastrointestinal (GI) and respiratory disease. Diagnostic clues include hypogammaglobulinemia, absent Tregs, and the presence of mostly naïve B and T cells with reduced memory compartments. Sequencing of BCL10 is likely to confirm a diagnosis, although functional assessment of novel BCL10 variants may be needed to definitively link the variant to the clinical phenotype. At this time, since only a single patient has been described and he died at the age of three from respiratory failure, validated treatment options remain unclear. However, based on our understanding of BCL10 biology, an allogeneic hematopoietic stem cell transplant would be anticipated to restore immune function by normalizing BCL10 protein expression and function in cells of hematopoietic origin.

## MALT1

### Role of MALT1 in immunity

MALT1 paracaspase (also known as mucosa-associated lymphoid tissue lymphoma translocation protein 1) was first identified from MALT lymphomas possessing the chromosomal breakpoint t(11;18)(q21;q21) (128–131). This led to the formation of an oncogenic fusion protein of MALT1 with inhibitor of apoptosis (IAP2) called API2-MALT1. It was later found that API2-MALT1 was capable of interacting with BCL10 and potently inducing NF-κB activation (7).

MALT1 is a ~92 kDa protein, which consists of an N-terminal death domain (DD), three immunoglobulin-like domains (Ig), and a caspase-like (paracaspase) domain (Figure 1). As mentioned in the BCL10 section, MALT1 exists in a complex with BCL10 (7), and together they associate with a variety of CARD proteins in response to stimulation to form the family of CBM complexes. This makes MALT1 an important regulator of both innate and adaptive immunity. Initially believed to act mostly as a scaffold for the recruitment of other NF-κB signaling proteins (e.g., TRAF6), MALT1 is now appreciated to also have important proteolytic activity (at mostly arginine residues), allowing it to cleave substrates involved in the regulation of NF-κB, JNK, mTORC1, and more [reviewed in (114, 132)] (Figure 2). There are currently a total of ten validated MALT1 paracaspase substrates: A20 (133), BCL10 (134), CYLD (135), RelB (136), Regnase-1 (137, 138), Roquin-1/2 (138), MALT1 (139), HOIL1 (140–142), NIK (143), and LIMA1α (144), with more likely to be discovered. By cleaving these substrates, MALT1 can positively regulate canonical NF-κB (A20), JNK (CYLD), DNA binding of RelA and c-Rel (RelB), and mRNA stability (Regnase-1 and Roquin-1/2). However, MALT1 protease activity may also negatively regulate NF-κB activity (HOIL1). How MALT1 paracaspase activity fine-tunes immune function in different cellular contexts is an area of intense research activity.

In order to better understand the physiological roles of MALT1, two important murine models have been generated: the *Malt1*^−/−^ mouse and the MALT1 paracaspase dead/mutated mouse (*Malt1*^*PD*/*PD*^) (Table 1). *Malt1*^−/−^ mice share many features with *Card11*^−/−^ and *Bcl10*^−/−^ mice, including having generally normal total numbers of B and T cells, diminished innate B cells (MZ and B1), severely impaired Treg numbers, and panhypogammaglobulinemia paired with compromised T-dependent antibody responses (16, 88–91). In response to stimulation, both B and T cells proliferate poorly, with T cells being more profoundly impacted as measured by the activation of NF-κB, JNK, p38, and the upregulation of activation markers. In addition, *Malt1*^−/−^ mice had absent germinal center B cells with an associated decrease in T follicular helper (Tfh) cells. Together, these studies confirmed that MALT1 is an essential regulator of T cell activation and Treg development. The contribution of MALT1 to B cell activation remains less clear.

*Malt1*^*PD*/*PD*^ mice on the other hand, had intact MALT1 protein expression and scaffolding activity, but abrogated paracaspase function (89, 91–93). Surprisingly, these mice developed spontaneous multi-organ inflammation, including autoimmune gastritis, which was not seen in MALT1-deficient mice. Interestingly, immune findings between the two mouse models were quite similar, including decreased MZ, B1 cells, Tregs, and proliferation, although these phenotypes were less pronounced in *Malt1*^*PD*/*PD*^ mice. In contrast to its scaffolding function, protease activity was mostly dispensable for NF-κB and JNK activation. Interestingly, *Malt1*^*PD*/*PD*^ mice possessed expanded Th1, Th2, Th17 phenotypes, CD4^+^ and CD8^+^ effector T cells, and elevated IFN-γ, IgE, and IgG. These studies highlighted the unique contributions of MALT1 scaffolding and paracaspase functions in signaling and lymphocyte development; for example, IKK and JNK activation were dependent on the scaffolding role rather than protease activity. In particular, it seems proteolytic activity is important for the development of anti-inflammatory Tregs as well as controlling excessive IFN-γ secretion and accumulation of effector T cells (93). More studies are needed to understand the exact factors and cell populations mediating this inflammatory phenotype. It is possible that this inflammatory phenotype may be mediated in part by the lack of Tregs and the inability of MALT1 to cleave HOIL1 to turn off NF-κB activation (114).

#### Loss-of-function MALT1 mutations causing combined immunodeficiency

Germline loss-of-function mutations in MALT1 cause CID (OMIM 615468). To date, six cases of MALT1 deficiency have been reported (21–24) (Figure 1 and Table 7). This includes a 4-year-old girl and 2-year-old boy who were both homozygous for the p.Ser89Ile mutation (21), a 15-year-old Kurdish-Canadian girl homozygous for the p.Trp580Ser mutation (22), a 1-month-old boy who was compound heterozygous for c.[1019-2A>G];[1060delC] mutations (23), and a 4-year-old boy and 7-year-old girl who were both homozygous for the p.Asp184Tyr mutation (24). All patients were identified by next generation sequencing techniques; the majority were found by WES (5/6 patients) and one case was discovered by whole genome sequencing (21). Most patients were born to consanguineous parents (4/6 patients) and possessed homozygous mutations, while one patient possessed *de novo* compound heterozygous mutations (23). These mutations span the length of the MALT1 protein, including the DD (21), the first Ig-like domain (24), the paracaspase domain (23), and the third Ig-like domain (22) and generally caused no MALT1 protein to be expressed (Figure 1).

**Table 7:** Clinical and laboratory phenotype of human MALT1 deficiency (LOF *MALT1* mutations).

**Mutation**	**p.Trp580Ser**	**p.Ser89Ile**	**p.Ser89Ile**	**c.[1019-2A>G];[1060delC]**	**p.Asp184Tyr**	**p.Asp184Tyr**
Age	15yo	4yo	2.25yo	9–13 months	7yo	4yo
Sex	F	F	M	M	F	M
Ethnicity	Kurdish	Lebanese	Lebanese	American	ND	ND
Consanguinity	+	+	+	–	+	+
Functional Impact	Loss	Loss	Loss	Loss	Loss	Loss
Inheritance	AR	AR	AR	AD	AR	AR
Gene Expression	Normal	Normal	ND	Decreased/none	Normal	Normal
Protein Expression	↓*↓↓*	None	ND	None	None	None
**INFECTIONS**						
**Pulmonary**	+	+	+	+	+	+
*Staphylococcus aureus*	+	–	+	–	–	–
*Streptococcus pneumoniae*	+	+	–	–	+	–
*Haemophilus influenzae*	–	–	+	–	–	–
*Klebsiella pneumoniae*	–	–	+	–	–	–
*Pneumocystis jirovecii*	–	–	–	–	+	–
*Pseudomonas*	–	+	–	–	–	–
CMV	+	–	–	+	+	–
EBV	–	–	–	–	+	–
RSV	–	–	–	+	–	–
Adenovirus	–	–	–	–	+	+
*Candida albicans*	–	+	–	–	–	–
**Gastrointestinal**	–	+	+	+	+	+
*Salmonella*	–	–	–	–	+	+
*Campylobacter jejuni*	–	–	–	–	+	–
*Clostridium difficile*	–	–	–	+	–	–
CMV	–	–	–	–	+	–
EBV	–	–	–	–	+	–
Rotavirus	–	–	–	–	+	–
Adenovirus	–	–	–	–	+	–
*Candida albicans*	–	–	+	–	–	–
**Skin**	+	–	–	+	+	+
*Staphylococcus aureus*	+	–	–	+	–	–
*Pseudomonas*	–	–	–	–	–	+
HSV-1	+	–	–	–	+	+
VZV	+	–	–	–	–	–
*Candida albicans*	–	–	–	+	+	+
**Blood**	–	–	–	+	+	–
*Staphylococcus aureus*	–	–	–	–	+	–
*Streptococcus pneumoniae*	–	–	–	–	+	–
CMV	–	–	–	+	–	–
**Urine**	–	+	+	–	–	–
CMV	–	+	+	–	–	–
**Meningitis**	–	+	–	–	–	–
*Haemophilus influenzae*	–	+	–	–	–	–
*Streptococcus pneumoniae*	–	+	–	–	–	–
**Keratitis**	–	–	–	–	+	–
HSV-1	–	–	–	–	+	–
**CLINICAL MANIFESTATIONS**						
Failure to thrive	+	+	+	+	+	–
Dysmorphic facies	+	–	–	–	+	+
Periodontal disease	+	+	+	+	+	+
Eczema	+	–	–	+	+	+
Enteropathy	+	+	+	+	+	–
Bronchiectasis	+	+	+	–	+	–
**THERAPY**						
**Transplantation**	+	–	–	+	+	+
Successful	+	N/A	N/A	+	+	+
**IVIG**	–	+	+	+	+	–
**Anti-microbial prophylaxis**	ND	ND	ND	+	+	+
Antibiotics	ND	ND	ND	+	+	+
TMP/SMX	ND	ND	ND	ND	+	+
**ANTI-VIRAL**						
Gancyclovir	–	–	–	+	–	–
Foscarnet	–	–	–	+	+	+
Acyclovir	–	–	–	–	+	+
**IMMUNOSUPPRESSION**						
**Pre-transplantation**	+	–	–	–	+	+
Treosulfan	–	–	–	–	–	–
Fludarabine	+	–	–	–	+	+
Busulfan	–	–	–	–	+	+
Alemtuzumab	+	–	–	–	+	+
Cyclophosphamide	–	–	–	+	–	–
Melphalan	–	–	–	+	–	–
R α-thymocyte globulin	–	–	–	+	–	–
**Post-transplantation**	+	–	–	+	+	+
Cyclosporine	+	–	–	+	+	+
Mycophenolate mofetil	+	–	–	–	+	+
Methylprednisone	+	–	–	–	–	–
Methotrexate	–	–	–	+	–	–
Tacrolimus	–	–	–	–	–	+
**Outcome**	Alive	Dead	Dead	Alive	Alive	Alive
**Cause of death**	N/A	Respiratory failure	Respiratory failure	N/A	N/A	N/A
**LYMPHOCYTES**						
**Total CD19 B cells**	↓	Normal	↓	Normal	Normal	Normal
Naive	↑	ND	ND	ND	ND	ND
(Class-switched) memory	↓	ND	ND	ND	ND	ND
MZ	↓	ND	ND	ND	ND	ND
**Total CD3 T cells**	↑	Normal	Normal	↑	↑	↑
CD4	↑	Normal	↑	↑	↑	↑
CD4 CD45RA	ND	Normal	Normal	Normal	Normal	↑
CD8	ND	↑	Normal	↑	Normal	↑
CD8 CD45RA	ND	ND	ND	↑	ND	ND
Treg	Normal	ND	ND	↓	↓	↓
**PROLIFERATION**						
PHA	↓	↓	↓	↓	↑	Normal
ConA	ND	↓	↓	↓	ND	ND
PWM	ND	↓	↓	Normal	ND	ND
CD3	ND	↓	↓	ND	ND	ND
CD3+CD28	ND	ND	ND	ND	↓	↓
Tetanus	ND	↓	↓	ND	ND	ND
Diphtheria	ND	↓	↓	ND	ND	ND
*Candida*	ND	↓	↓	ND	ND	ND
**IMMUNOGLOBULINS**						
IgG	Normal	Normal	Normal	↓	Normal	Normal
IgA	Normal	Normal	Normal	Normal	Normal	Normal
IgM	Normal	Normal	Normal	↓	↓	↓
IgE	↑	Normal	Normal	Normal	↑	↑
**Specific antibodies**						
Tetanus	Positive	Negative	Negative	Negative	ND	ND
Pneumococcal	ND	Negative	Negative	Negative	ND	ND
Diphtheria	Positive	ND	ND	Negative	ND	ND
Isohemagglutinins	Positive	Negative	Negative	↓	ND	ND
*Haemophilus influenzae B*	ND	ND	ND	Negative	ND	ND
References	(22)	(21)	(21)	(23)	(24)	(24)

*Summary of molecular, immunological, infectious, and pathological findings for all MALT1-deficient patients described to date. ND, no data; ↑, increased levels relative to normal range; ↓, decreased levels relative to normal range; +, present; −, absent; EBV, Epstein-Barr virus; HSV-1, herpes simplex encephalitis 1; VZV, varicella-zoster virus; R, rabbit*.

MALT1 deficiency is characterized by recurrent sinopulmonary infections, enteropathy, eczema, periodontal disease, and failure to thrive (6). Indeed, patients typically presented with recurrent bacterial, viral, and fungal infections affecting the lungs (6/6 patients), skin (3/6 patients), and GI tract (3/6 patients) (Table 7). However, some patients experienced bloodborne infections, including one patient who had *Staphylococcus aureus* and *Streptococcus pneumoniae* bacteremia (24) and another who had CMV viremia (23). One of the patients also had meningitis positive for *Streptococcus pneumoniae* and *Haemophilus influenzae* (21).

Periodontal disease (6/6 patients) was common to all patients, with many developing aphthous ulcers, cheilitis, gingivitis, and thrush (21–24, 145). In addition, both dermatitis (4/6 patients) and inflammatory GI disease (5/6 patients) were frequently reported findings. Consequently, significant T cell infiltration in the skin and or the GI tract was found in biopsies (21–24). Developmentally, half of the patients had abnormal facial features (although these may be related to inflammatory changes affecting the oral cavity) (22, 24) and the majority had failure to thrive (5/6 patients).

Some patients had additional unique presentations of disease. The patient carrying the p.Trp580Ser was found to have very low bone density and suffered from fractures due to low-impact injuries (22). She also recurrently generated granulation tissue on her vocal cords, larynx, and external auditory canal. In addition, the two patients carrying the p.Ser89Ile mutation also developed mastoiditis (21).

Immunophenotyping of these MALT1-deficient patients found generally normal (21, 23, 24) or decreased (21, 22) B cell numbers. Interestingly, in contrast to other patients, the p.Trp580Ser mutation was associated with a developmental block in their B cell compartment characterized by absent MZ B cells, reduced transitional and class-switched memory B cells, and elevated naïve B cells (22). Despite relatively normal B cell populations, only the p.Ser89Ile siblings had normal serum immunoglobulin titers (21), while half of the patients possessed diminished IgM (23, 24) and elevated IgE (22, 24). On the other hand, CD3^+^ and CD4^+^ T cells were found to be expanded in most patients (4/6 patients) with the exception of the p.Ser89Ile siblings (21) who were within the normal range. CD8^+^ T cells were mostly elevated (3/5 patients) or within the normal range. Similar to CARD11- and BCL10-deficient patients, these patients also generally had diminished Tregs (3/4 patients). All MALT1-deficient patient T cells showed impaired proliferation in response to PHA or α-CD3/CD28 stimulation. In line with impaired T cell responses, most patients also possessed poor vaccine antibody titers (3/4 patients) (21–23).

Biochemical characterization of patient cells demonstrated completely abrogated NF-κB phosphorylation and/or IκBα degradation, along with diminished IL-2 secretion (21–24). In addition, McKinnon et al. was also able to demonstrate impaired paracaspase activity as measured by BCL10 cleavage (22). Using these MALT1-deficient patient cells, the same group discovered the novel MALT1 substrate HOIL1 (140). This defined a novel negative regulatory role for MALT1 in NF-κB signaling, where, by cleaving HOIL1, linear ubiquitination-mediated signaling and inflammation is decreased/turned off. It is possible that in MALT1-deficient patients, the loss of MALT1 proteolytic activity on HOIL1 leads to an accumulation in linear ubiquitination, resulting in unrestricted NF-κB activation and chronic inflammation, thus contributing to the exaggerated skin and mucosal inflammation seen in MALT1-deficient patients (140).

#### Diagnosis and treatment of MALT1 deficiency

All cases of MALT1 deficiency described to date have been discovered by next generation sequencing. However, based on this small cohort of patients, there are some diagnostic clues that raise suspicion for MALT1 deficiency (Table 8). Specifically, MALT1 deficiency should be considered in patients who present with the majority of the following: (i) severe recurrent sinopulmonary infections positive for bacteria or viruses, (ii) severe inflammatory GI disease, (iii) eczematous rash, (iv) severe periodontal disease, and (v) failure to thrive. Diagnostic testing “red flags” include finding relatively normal lymphocyte and B cell numbers, expanded CD3^+^ and CD4^+^ T cell subsets, impaired T cell proliferation, and compromised NF-κB phosphorylation, IκBα degradation, and IL-2 secretion.

**Table 8:** “Red flags” suggestive of human MALT1 deficiency.

**Clinical features**	**Proportion**	**Penetrance (%)**
**INFECTIONS (BACTERIAL/VIRAL/FUNGAL)**		
Pulmonary	6/6	100
Skin	3/6	50
Gastrointestinal tract	3/6	50
**ORGAN INVOLVEMENT**		
Periodontal disease	6/6	100
Gastrointestinal inflammation	5/6	83
Dermatitis	4/6	67
**DEVELOPMENT**		
Failure to thrive	5/6	83
Abnormal Facies	3/6	50
**LABORATORY TESTS**		
**Cell populations**
Normal lymphocytes numbers	3/4	75
↓ Treg	3/4	75
Normal B cells	4/6	67
↑ CD3^+^, CD4^+^ T cells	4/6	67
↑ CD8^+^ T cells	3/5	60
**Response**		
↓ T cell proliferation (PHA, α-CD3/CD28)	6/6	100
↓ NF-κB phosphorylation/IκBα degradation	6/6	100
↓ IL-2 secretion	6/6	100
**Ig**		
↓ specific antibodies	3/4	75
↓ IgM	3/6	50
↑ IgE	3/6	50

*Common features found in MALT1-deficient patients. Included are proportion of patients where data is available and the percentage of patients with that finding. ↑, increased levels relative to normal range; ↓, decreased levels relative to normal range*.

MALT1 deficiency can be cured by hematopoietic stem cell transplantation (4/6 patients received successful transplants) (23, 24, 146). Highlighting the value of curative transplantation, the siblings homozygous for the p.Ser89Ile were not transplanted and they continued to experience persistent infections until their eventual deaths due to respiratory failure at the ages of 7 and 13.5 years (21). Successful donor choices have included: bone marrow from an HLA-matched sibling (p.Trp580Ser patient) (146); peripheral stem cells from two unrelated 10/10 HLA-matched donors (p.Asp184Tyr siblings) (24); and peripheral stem cells from an unrelated 9/10 HLA-matched donor (c.[1019-2A>G];[1060delC] patient) (23). Despite successful engraftment being achieved in these patients, there were some noteworthy post-transplantation complications. The p.Trp580Ser patient experienced a range of infections including CMV, Epstein-Barr virus (EBV), VZV, and herpes simplex virus-1 (HSV-1), adenovirus viremia, *Staphylococcus aureus* bacteremia, *Klebsiella pneumoniae* pneumonia, extended spectrum beta-lactamase positive *Streptococcus pneumoniae* and *Escherichia coli*, BK virus-associated hemorrhagic cystitis, and rotavirus-associated gastroenteritis (146). The c.[1019-2A>G];[1060delC] patient developed diarrhea and CMV viremia (23). The p.Asp184Tyr siblings developed transient CMV viremia and the younger brother developed an adenovirus infection as well as bacterial pneumonia (24).

## CBM mutations in relation to other primary atopic TCR-mediated disorders

Germline MALT1 and CARD11 mutations can be considered primary atopic disorders in that they are associated with early-onset, severe atopic disease, amongst other comorbidities (99). Primary atopic disorders are often associated with primary immunodeficiency, most commonly caused by mutations in cytokine signaling or disruptions in TCR signaling or repertoire. Atopy is hypothesized to be caused by the propensity of naïve CD4 T cells to skew toward Th2 differentiation when relatively weak TCR signals are delivered (147). While CBM-associated mutations are the most directly linked to TCR signaling, other disruptions which can indirectly impact TCR signaling to cause atopic disease include actin cytoskeleton remodeling genes such as dedicator of cytokinesis 8 (DOCK8) deficiency (148), Wiskott-Aldrich Syndrome (WAS) protein interacting protein (WIP) deficiency (149), Wiskott-Aldrich Syndrome (150), actin related protein 2/3 (ARP2/3) complex mutations (151–153) and, potentially, ZAP70 deficiency (154, 155). The clinical presentation of DOCK8 deficiency is somewhat similar to CBM mutation-associated atopy, though infectious and neoplastic manifestations are more severe. WIP-, WAS- and ARP2/3-associated disease have more systemic manifestations including thrombocytopenia likely due to more broad protein expression patterns. In addition, the clearest TCR repertoire defect associated with atopic disease is Omenn syndrome, which is caused by hypomorphic mutations in most genes associated with SCID [including one CARD11-deficient case (19)], and results in an oligoclonal expansion of CD4 T cells. This, in turn, leads to severe dermatitis, elevated IgE, eosinophilia, and lymphoproliferation.

## Comparing and contrasting CBM-opathies: unanswered questions

In adaptive immunity, the CBM complex functions in a highly synergistic manner. In line with this, CARD11-, BCL10-, and MALT1-deficient patients share many features, including having CID/SCID with normal total B and T cell numbers, aberrant B and T cell subsets, little-to-no Tregs, impaired T cell proliferation, and recurrent bacterial/viral infections (17–24). As a group, patients with these CBM-opathies have established that the CBM complex is a critical regulator of human Treg development and tolerance; however, the exact mechanisms by which this occurs are not completely understood. In murine models, it is thought that the ability of the CBM complex to modulate TCR signal strength and transduce signals downstream of the IL-2R contribute significantly to this process (33, 88, 89, 92, 93, 156).

**Figure 3:**
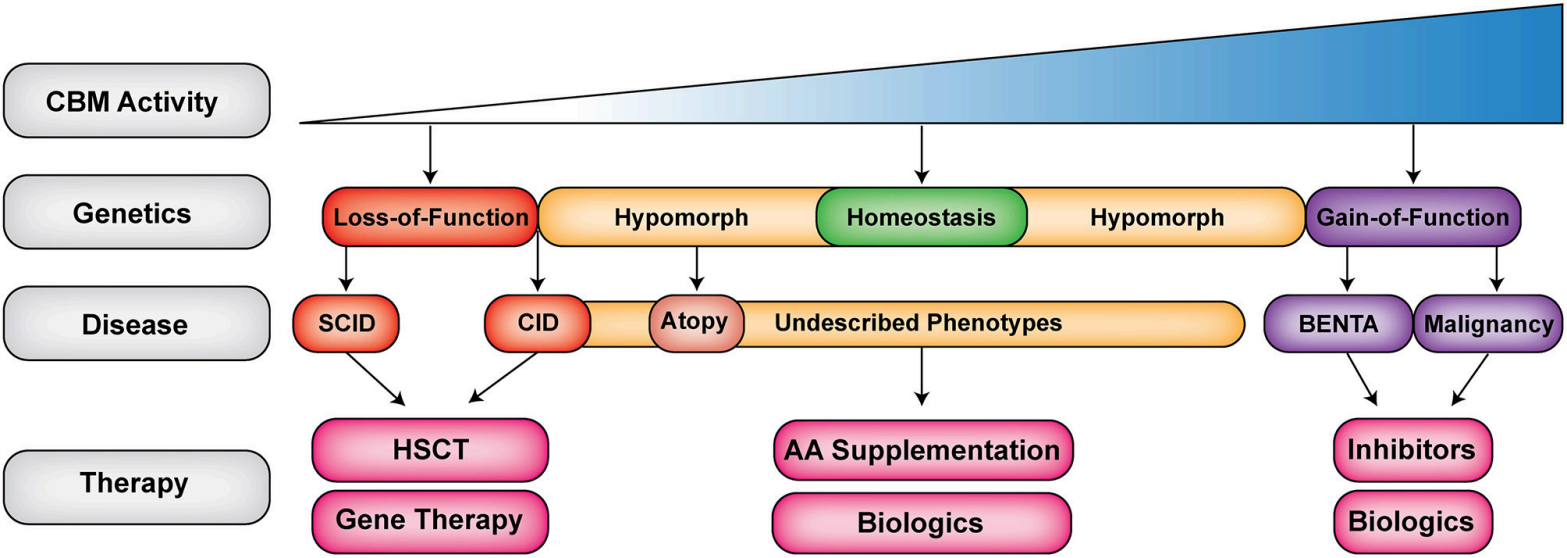
The expanding clinical spectrum of CBM-opathies. Shown is a gradient of CBM activity caused by germline mutations. Activity ranges from absent to hyperactive CBM activity. Red indicates loss-of-function (LOF) mutations (CARD11, BCL10, and MALT1 deficiencies), yellow indicates hypomorphic mutations that do not completely abrogate signaling leading to combined immunodeficiency and atopy as well as novel emerging phenotypes (DN LOF *CARD11*), purple indicates gain-of-function (GOF) mutations that can lead to BENTA (GOF *CARD11*) or malignancy (somatic GOF in CBM). Biologics refer to antibodies, which target cells, cytokines or cell surface receptors. SCID, severe combined immunodeficiency; CID, combined immunodeficiency; AA, amino acid; HSCT, hematopoietic stem cell transplantation.

While CARD11 is mostly restricted to hematopoietic cells, BCL10 and MALT1 have much broader cellular expression and associate with other CARD proteins downstream of a diverse assortment of receptors (66). Thus, individual CBM-opathies each have their own unique features (Figure 3). For example, CARD11-deficient patients characteristically exhibit panhypogammaglobulinemia, which is not present in MALT1 or BCL10 deficiency. In contrast, all CBM-deficient mice have panhypogammaglobulinemia. This demonstrates both important differences between mouse models and human patients as well as an incomplete understanding of how the CBM complex regulates antibody production. Another clinical feature that varies between the CBM-opathies is susceptibility to *Pneumocystis jirovecii* pneumonia (PJP). PJP is a very common infection in CARD11 deficiency (reported in 75% of identified patients) but is not a reported pathogen in MALT1 and BCL10 deficiency. It is still not known why *Pneumocystis jirovecii* seems to preferentially infect CARD11-deficient patients.

Moving beyond infections, both MALT1- and BCL10-deficient patients presented simultaneously with immunodeficiency and immune dysregulation, where in addition to recurrent infections, they also developed inflammatory gastrointestinal disease (20–24). These features were not present in CARD11-deficient patients, nor were they found in *Card11*^−/−^, *Malt1*^−/−^, and *Bcl10*^−/−^ mice. In fact, the pharmacological inhibition of MALT1 in dextran sulfate solution (DSS)-induced colitis was found to be protective through the inhibition of NF-κB and NLRP3 inflammasome activation in macrophages (157), and a T cell-dependent transfer model of autoimmune colitis found that *Malt1*^−/−^ T cells were unable to induce colitis (92). However, it is important to note that *Malt1*^*PD*/*PD*^ mice developed inflammatory gastrointestinal disease, and this was associated with an expansion of Th1, Th2, and Th17, with increased inflammatory cytokines and IgE, which was not present in the *Malt1*^−/−^ mice (89, 91–93). It is tempting to speculate that the diminished Tregs, dysregulated tolerance, and lack of MALT1 paracaspase regulation collectively mediated the inflammatory phenotype in patients. In addition, both MALT1-deficient and BCL10-deficient patients displayed elevated CD3^+^ and CD4^+^ T cells, but it is not known whether there was any skewing in Th1 and Th2 responses (as is the case in *Malt1*^*PD*/*PD*^ mice), which could be significant pro-inflammatory cytokine secretors, thus contributing to pathology. It should also be noted that MALT1 and BCL10 have wider expression than CARD11 and some non-hematopoietic cells in these patients could be contributing to the gastrointestinal pathology.

GOF and DN LOF *CARD11* mutations give rise to considerably different phenotypes from CBM-deficiencies, with GOF manifesting chiefly as selective B cell lymphocytosis and DN LOF leading to severe atopic disease. Deeper mechanistic studies are needed to address several outstanding questions, including (i) how GOF *CARD11* mutations dampen T cell responsiveness, (ii) whether GOF *CARD11* mutations enhance JNK and mTORC1 signaling, and how this contributes to differential T and B cell responses, (iii) if/how DN *CARD11* mutations ultimately skew Th2 responses via decreased NF-κB signaling and/or restricted CARD11-dependent glutamine uptake, and (iv) whether DN *CARD11* mutations affect B cell intrinsic signaling, including the fate of class-switched IgE^+^ B cells. Overall, shared phenotypes in BENTA and CADINS patients (e.g., poor antibody responses, increased respiratory and skin infections) emphasize the requirement for properly “tuned” CBM signaling to ensure proper B and T cell differentiation in response to antigens in order to maintain immune homeostasis.

## Novel therapeutic insights emerging from our understanding of human CBM-opathies

CBM-opathies have been invaluable in enhancing our understanding of how dysregulated CBM complex signaling contributes to the pathogenesis of various diseases including immunodeficiency, atopic disease (94), autoimmunity (158), and malignancies (159). Given the role of the CBM complex in a range of human pathologies, there is considerable interest in developing and studying therapeutics that can target/ameliorate these diseases. In the realm of cancer, targeting either the CBM complex or the catalytic function of MALT1 have been the methods of choice (160). In particular, MALT1 inhibitors have received a great deal of attention for their specificity and efficiency. These inhibitors may eventually be promising options for treating cancers and diseases that have a lymphoproliferative component, including BENTA (104). However, given the central position of the CBM complex in signaling, inhibition should be approached with caution. Here, LOF mutations in individual CBM components and the recent characterization of *Malt1*^*PD*/*PD*^ mice have been uniquely informative in highlighting possible side effects that can arise from the therapeutic inhibition of the CBM complex, including decreasing Tregs and tolerance (114).

Currently, the treatment of complete CARD11, BCL10, and MALT1 deficiencies relies upon hematopoietic stem cell transplantation in order to functionally normalize immune function (with immunoglobulin replacement and prophylactic antimicrobials used as supportive therapy). Without transplantation, the survival rate is very low. Moving forward, autologous gene therapy may be an attractive therapeutic option, whereby patient hematopoietic stem cells could be “corrected” by genetic approaches (e.g., viral transduction or CRISPR/Cas9 editing) and re-infused to give rise to a normal immune system (161). In support of this approach, transplantation outcomes have been quite good for CBM deficiency patients (17, 146) and the artificial expression of WT genes in patient cells is able to rescue NF-κB activation (22). Further proof of concept studies in mice or patient stem cells will have to be done to determine efficacy and safety.

The initial description of *Card11*^*un*/*un*^ mice (13) paired with the recent discovery of DN LOF mutations in CARD11 causing atopy and immunodeficiency (31, 32), implicated the CBM complex in the pathogenesis of allergic disease. Affected patients were found to have decreased upregulation of ASCT2 and impaired mTORC1 signaling, which is thought to contribute to Th2 skewing (32). Since it was previously shown that impaired mTORC1 signaling and Th1 differentiation could be rescued by glutamine supplementation (64), Ma et al. tested whether glutamine supplementation could rescue the phenotype of patient cells. Interestingly, this was able to partially rescue signaling defects (32). This demonstrated that modulating immune metabolism through amino acid supplementation could be useful for therapy. Indeed, glutamine supplementation is currently being explored in low birth weight infants for the reduction of atopic dermatitis and has shown some success (162).

## Concluding remarks

The CBM complex is an essential molecular bridge linking cell surface antigen receptor signaling with downstream activation of NF-κB, JNK, and mTORC1. This makes it a critical regulator of lymphocyte activation, differentiation, proliferation, maintenance, and metabolism. Since the discovery of germline loss-of-function mutations in CARD11 causing SCID just 5 years ago (17), ~48 patients with genetically confirmed CBM-opathies have been described. Germline mutations in this complex have led to an impressive spectrum of diseases, ranging from CID/SCID to CID with atopy to BENTA disease (Figure 3). The detailed study of these rare patients with CBM-opathies has provided unique insights into how the CBM complex regulates human immune reactivity and tolerance. Ultimately, the discovery and characterization of more CBM-opathies will not only benefit the affected patients but will broadly inform any future therapeutic targeting of these signaling pathways in cancer, autoimmunity, and allergic disease.

## Author contributions

All authors listed have made a substantial, direct and intellectual contribution to the work, and approved it for publication.

### Conflict of interest statement

The authors declare that the research was conducted in the absence of any commercial or financial relationships that could be construed as a potential conflict of interest.
